# Valorization of Glucose-Derived Humin as a Low-Cost, Green, Reusable Adsorbent for Dye Removal, and Modeling the Process

**DOI:** 10.3390/polym15153268

**Published:** 2023-07-31

**Authors:** Thakshila Nadeeshani Dharmapriya, Ken-Lin Chang, Po-Jung Huang

**Affiliations:** 1 Institute of Environmental Engineering, College of Engineering, National Sun Yat-sen University, Kaohsiung 80432, Taiwan; 93thakshilanadee@gmail.com (T.N.D.); klchang@mail.nsysu.edu.tw (K.-L.C.); 2 Department of Chemical and Materials Engineering, National Central University, Taoyuan 320317, Taiwan

**Keywords:** glucose-derived humin, dye removal, response surface methodology, adsorption, polymeric furanic-type structure

## Abstract

Glucose can be isomerized into fructose and dehydrated into key platform biochemicals, following the “bio-refinery concept”. However, this process generates black and intractable substances called humin, which possess a polymeric furanic-type structure. In this study, glucose-derived humin (GDH) was obtained by reacting D-glucose with an allylamine catalyst in a deep eutectic solvent medium, followed by a carbonization step. GDH was used as a low-cost, green, and reusable adsorbent for removing cationic methylene blue (MB) dye from water. The morphology of carbonized GDH differs from pristine GDH. The removal efficiencies of MB dye using pristine GDH and carbonized GDH were 52% and 97%, respectively. Temperature measurements indicated an exothermic process following pseudo-first-order kinetics, with adsorption behavior described by the Langmuir isotherm. The optimum parameters were predicted using the response surface methodology and found to be a reaction time of 600 min, an initial dye concentration of 50 ppm, and a GDH weight of 0.11 g with 98.7% desirability. The MB dye removal rate optimized through this model was 96.85%, which was in good agreement with the experimentally obtained value (92.49%). After 10 cycles, the MB removal rate remained above 80%, showcasing the potential for GDH reuse and cost-effective wastewater treatment.

## 1. Introduction

Lignocellulosic biomass is regarded as a promising alternative feedstock for renewable transportation biofuels and bio-based chemicals [1,2]. Depolymerizing cellulose and hemicellulose in a lignocellulosic biorefinery yield hexose (C6-glucose, fructose) and pentose units (C5-xylose). These monosaccharides can be further converted into bio-chemicals and biofuels [3,4,5,6,7]. This process involves the formation of significant amounts of a black and uncontrollable material known as humin. Humin is a carbonaceous, heterogeneous, and polydisperse macromolecule [8]. Several studies have identified humins as diverse polymers consisting of furan rings connected by aliphatic carbon bonds and various oxygen-containing groups. These humins are categorized as furanyl polymers, resulting from the polymerization of sugar, HMF (hydroxymethylfurfural), or FF (furfuryl alcohol), along with active intermediate compounds [8,9,10,11]. The yield of humin is heavily influenced by feedstock and process parameters such as reaction medium, temperature, reaction time, pH, etc. In the aqueous phase, humin selectivity can reach 50% on a carbon basis. Recently, in the bio-refining process using alkyl-phenol as the organic solvent, humin synthesis has been shown to reach 25–45 wt%. Consequently, the formation of humin reduces the yield of other valuable products such as 5-HMF, FF, levulinic acid (LA), etc. Therefore, it is crucial to valorize the byproduct humin to ensure the economic and environmental viability of the entire biomass conversion process [1,3,4].

Despite humin being a known substance for an extended period, its primary applications have remained confined to energy and heat applications. However, the escalating production of furans platform chemicals has sparked a surge of interest in exploring additional value-added applications for humin beyond combustion [1]. These applications encompass its potential as a carbonaceous source for gas synthesis [12], its liquefaction into alkyl phenolics and higher oligomers using a mixture of formic acid/2-propanol with supported ruthenium catalysts [13], its combination with biomass-derived humin and poly-furfuryl alcohol (PFA) for composite elaboration [14], its enhancement of the modulus and tensile strength of pure PFA resins [15], and the synthesis of humin-based iron oxide catalytic nanocomposites etc. [16]. Moreover, humin extracted from soil or bio-refinery processes has been effectively utilized as an adsorbent to remove heavy metals such as Cd, Cr, Pb, and Al from aqueous media [17,18,19,20,21].

Recent developments in unconventional solvents led to deep eutectic solvents (DES) formed by combining a hydrogen-bond donor (HBD) with a hydrogen-bond acceptor (HBA) [22,23]. DESs consist of safe, affordable, and biodegradable components such as choline chloride (ChCl), carbohydrates, carboxylic acids, and glycerol [22,24]. These DES mixtures have lower melting points than individual components [25,26]. DESs share physicochemical traits with ionic liquids (ILs), being non-flammable, low in volatility, and suitable for long-term recycling [24]. Past experiments aimed at creating glucose-based deep eutectic solvents (DES) using ChCl and the monosaccharide sugar D-glucose anhydrous. Analysis of their physical properties indicated the potential for various industrial applications, including mediums for chemical reactions [27].

Considerable amounts of organic dyes find applications in industries such as paper, apparel, textiles, dyestuffs, leather, plastics, etc., leading to health problems for humans and animals, along with environmental pollution. These dyes tend to absorb and reflect sunlight, reducing sunlight penetration into water bodies, resulting in reduced algae activity and dissolved oxygen levels [28,29,30,31]. Among these dyes, Methylene Blue (MB) stands out as a highly consumed cationic dye utilized in biological, chemical, and medical applications [21]. MB is an aromatic heterocyclic basic dye with a molecular weight of 319.85 g mol^−1^ and a λ_max_ of 664 nm [32,33,34], being highly water-soluble and stable in solution at room temperature [34]. According to the International Union of Pure and Applied Chemistry (IUPAC), the chemical name of MB is [3,7-bis(dimethylamino) phenothiazine chloride tetramethylthionine chloride], and its color index (CI) is 52,015 [35,36]. It exhibits a deep blue color when oxidized and becomes colorless when reduced [37]. However, MB is toxic, non-biodegradable, and carcinogenic, posing risks to human health and the environment [38,39]. Another dye, Methyl Orange (MO), has a molecular weight of 327.33 g mol^−1^ and a λ_max_ of 465 nm. Its IUPAC name is sodium 4-[(4-dimethylamino) phenyldiazenyl] benzenesulfonate. MO is an anionic azo dye that is water-soluble, non-biodegradable, toxic, and considered carcinogenic [40,41].

Biodegradable dyes are a category of dyes that can be broken down naturally by microorganisms into simpler, non-toxic substances over time [42]. Unlike conventional synthetic dyes, which are typically derived from petrochemicals and pose significant environmental concerns due to their non-biodegradable nature and potential toxicity, biodegradable dyes offer a more sustainable and eco-friendlier alternative [43]. Additionally, advancements in biotechnology have enabled the synthesis of biodegradable dyes through environmentally benign methods, reducing the need for harmful chemicals in their creation [44,45]. For example, Lin et al. studied sustainable wool yarn dyeing using blends of madder red (MR), gardenia blue (GB), and gardenia yellow (GY) dyes to create diverse color shades (color triangle) at different dye mass ratios, eliminating the need for mordants (metallic salts) [46].

Promising technologies have been researched to eliminate synthetic and non-biodegradable dyes from the environment. Among these, adsorption stands out due to its simple design, ease of use, and cost-effectiveness for reusability. Yet, few studies have examined the use of soil-extracted humin as an economical adsorbent for removing dyes from water [29,47,48]. Furthermore, to the best of our knowledge, there is no previous evidence of utilizing glucose-derived humin (GDH), a byproduct from bio-refinery processes, for dye adsorption. GDH, possessing a polymeric furanic-type structure, was synthesized through the reaction of D-glucose with an allylamine catalyst in a DES medium, followed by a carbonization step. Subsequently, its efficacy in adsorbing organic MB dye was tested. The adsorption process was assessed concerning various factors, such as pH, temperature, contact time, initial dye concentrations, adsorbent weight, and comparison with anionic dye MO. Furthermore, isotherm models were employed to understand the adsorption behavior, while reaction kinetics and thermodynamics were also investigated. To optimize the reaction parameters, response surface methodology was employed.

## 2. Materials and Methods

### 2.1. Materials

Allylamine (C_3_H_7_N, 98%) and choline chloride were [(CH_3_)_3_NCH_2_CH_2_OH]^+^Cl^−^ purchased from Alfa Aesar (Heysham, England). D-glucose (C_6_H_12_O_6_, 99.5%), methylene blue (C_16_H_18_N_3_ClS, 95%), and methyl orange (C_14_H_14_N_3_NaO_3_S, 85%), methanol (CH_3_OH, 99.9%) were purchased from Sigma-Aldrich (Burghausen, Germany). All chemicals used in this study were of analytical grade and used without further purification. All the solutions were made with high-purity water with 18 MΩ×cm resistance.

### 2.2. Synthesis and Collection of GDH Byproduct

To prepare GDH, the following procedure was employed: Choline chloride (ChCl), D-glucose, and deionized H_2_O were mixed in a 1:1:1 (w%) ratio in a round-bottom flask. An allylamine (AA) catalyst was added to D-glucose in a 5:1 (w%) ratio. The flask was placed in an oil bath, and reflux instrumentation was set up at 393 K (120 °C) with a stirring speed of 350 rpm for 12 h. After cooling to room temperature, the black-colored precipitate was separated from the medium, washed thoroughly with 2 L of DI water, and centrifuged at 10,000 rpm for 10 min to collect water-insoluble GDH. The collected GDH was dried at 378 K (105 °C) overnight and then ground into a powder. Subsequently, the pristine GDH was carbonized by heating it at 773 K (500 °C) in an oven for 2 h.

For studying the effect of different synthesis temperatures on pristine GDH’s surface area, pore size, and pore volume while keeping other parameters constant, GDH was synthesized at 353 K (80 °C), 373 K (100 °C), 393 K (120 °C), 413 K (140 °C), and 433 K (160 °C). The analysis was performed using the ASAP 2020 Plus instrument ASAP 2020 Plus instrument (Micromeritics Instrument Corporation, 4356 Communi-cations Drive Norcross, GA 30093, USA).

### 2.3. Characterization of GDH

The BET surface area and porous volume of the synthesized GDH were assessed using an ASAP 2020 Plus instrument (Micromeritics Instrument Corporation, 4356 Communications Drive, Norcross, GA 30093, USA). Nitrogen adsorption and desorption isotherms were measured at 77 K (350 °C) after degassing the GDH samples in a vacuum at 373 K (100 °C) for 12 h. To examine the GDH’s structure, infrared spectra were obtained using a Thermo Nicolet Is5 Fourier-transform infrared (FT-IR) spectrometer (Thermo Fisher Scientific, 168 Third Avenue, Waltham, MA USA 02451). The absorbance was measured with a resolution of 0.9 cm^−1^, and the FT-IR wavenumber ranged from 400 to 4000 cm^−1^. For material structure determination, powder X-ray diffraction (PXRD) measurements were conducted at room temperature using a Bruker D8 instrument; ADVANCE-XRD (Blue Scientific Limited, St. John’s Innovation Centre, Cowley Road, Cambridge, CB4 0WS). Additionally, the morphological features of the synthesized GDH were analyzed using a Schottky Field Emission Scanning Electron Microscope (SEM) SU5000 (Sukehiro Ito, Science & Medical Systems Design Division, Hitachi High Technologies Corporation, Tokyo, Japan).

### 2.4. Sample Reactor for Dye Adsorption and Analysis

The experiments were carried out using the PCX-50B Discover Multichannel Photochemical Reaction System (purchased from A08, 11/F, Changyin building, No. 88, Yongding Road, Haidian District, Beijing, China). The stirring speed was set at 300 rpm, and no light was applied, while the temperature-control system in the machine ensured that temperature variations did not impact the experimental outcomes. The typical experiments were conducted at 298 K (25 °C).

To quantitatively analyze the dyes, a UV-visible spectrophotometer (Hitachi U-2900/U-2910 UV-Vis Double Beam Spectrophotometer, Hitachi High Technologies America, Inc.) with a 10 × 10 mm cuvette holder and a 10 mm path length was used. The samples for analysis were diluted 300 times, and DI water served as the blank sample. The spectrophotometer scanned within a wavelength range of 200–800 nm. Calibration curves for the UV-visible spectrophotometer were generated using a series of standard dye solutions due to the ring structure and color of the dyes.

### 2.5. Use GDH as a Dye Adsorbent

To assess the removal efficiency of MB dye using carbonized GDH, a standard experiment was conducted. In this experiment, 0.1 g of GDH was introduced into a glass bottle containing a 25 ppm MB dye solution (initial concentration) and stirred at 300 rpm at room temperature for a duration of 10 h. Afterward, the samples were subjected to centrifugation to separate the supernatant. A UV-visible spectrometric analysis was then carried out to measure the remaining MB dye concentrations following the adsorption process. The following equations [Equations (1) and (2)] were used to calculate the adsorption capacity and removal efficiencies of the GDH adsorbent for MB dye,

(1) $$Q_{e}=\frac{\left(C_{o}-{\;C}_{e}\right)V}{W}$$

(2) $$\mathrm{Removal}\;\mathrm{Efficiency}=\frac{\left(C_{o}-{\;C}_{e}\right)}{C_{o}}\times 100$$

where Q_e_ (mg/g) = equilibrium adsorption capacity of the MB dye onto GDH adsorbent, C_o_ (mg/L) = initial concentration of aqueous MB dye solution, C_e_ (mg/L) = concentration of the aqueous MB dye solution after adsorption, V (L) = volume of the aqueous MB dye solution, and W (g) = mass of the GDH adsorbent [49,50,51,52].

### 2.6. Effect of Parameters on Dye Adsorption

Initially, MB dye adsorption experiments were conducted using both pristine GDH and carbonized GDH to compare their dye removal efficiencies. Subsequent experiments focused solely on carbonized GDH. Various adsorbent weights (ranging from 0.025 to 0.150 g) and initial concentrations of MB dye (ranging from 5 ppm to 50 ppm-5, 15, 25, 35, and 50 ppm) were utilized to identify the optimized GDH weight and initial concentration of the adsorbate, respectively.

To explore the impact of medium acidity, a series of 10 mL, 25 ppm MB aqueous solutions were prepared at pH levels of 1.00, 2.00, 4.00, 6.00, 8.00, and 10.00 using HCl and NaOH for pH adjustments. Full wavelength scan spectra were obtained using the UV-visible spectrophotometer for each MB dye solution at the specified pH ranges to determine the λ_max_ of MB dye before analyzing the remaining MB concentrations after the adsorption experiment. In each glass bottle, 0.1 g of GDH was added, and the dye adsorption conditions and analysis methods described in Section 2.5 were followed. Each sample was accompanied by a blank solution without a sorbent, and both were treated and analyzed in the same manner. Additionally, the same experimental procedure was conducted for MO dye.

### 2.7. Adsorption Kinetics and Thermodynamics

The adsorption control mechanism and potential rate-controlling steps of MB dye on GDH were assessed using two kinetic models: the pseudo-first order and the pseudo-second order models. The linear forms of the equations can be represented as Equations (3) and (4), respectively,

(3) $$\ln(Q_{e}-Q_{t})={\mathrm{lnQ}}_{e}-k_{1}t$$

(4) $$\frac{t}{Q_{t}}=\frac{1}{k_{2}Q_{e}^{2\;}}+\frac{1}{Q_{e}}$$

where Q_t_ (mg/g) was the amount of adsorbed dye at adsorption time t (min), k_1_ (min^−1^) and k_2_ (g/mg.min) were the rate constants of the pseudo-first-order and the pseudo-second-order, respectively [53]. The activation energy for dye adsorption onto the GDH was calculated by using the Arrhenius equation, which is indicated as Equation (5) [49,54].

(5) $$k_{2}=k_{0}.\exp^{\left(-\frac{E_{a}}{\mathrm{RT}}\right)}$$

The above equation can be linearized by taking logarithms as indicated in Equation (6),

(6) $$\ln k_{2}=\ln k_{0}-\frac{E_{a}}{\mathrm{RT}}$$

where k_o_ (g/mg.min) is the frequency factor, R (8.314 J·K^−1^·mol^−1^) is the universal gas constant, E_a_ (kJ mol^−1^) is the activation energy of the adsorption, and T (K) is the absolute temperature [51,54]. Adsorption experiments were conducted at 298, 308, 318, 328, and 338 K to determine the effect of temperature.

The thermodynamic parameters concerning the adsorption of MB dye, which include the standard free energy change (ΔG°), standard enthalpy change (ΔH°), and standard entropy change (ΔS°), were computed using the following method. Equation (7) provides the Gibbs free energy changes of the adsorption process, which are linked to the equilibrium constant [53].

(7) $${\Delta G}^{{}^{\circ}}=-{\mathrm{RTlnK}}_{c}$$

K_c_ can be calculated using Equation (8).

(8) $$K_{c}=\frac{C_{\mathrm{Ae}}}{C_{e}}$$

The values of ΔH° and ΔS° were obtained by analyzing the slope and intercept of the linear Van’t Hoff plot (Equation (9)).

(9) $${\mathrm{lnK}}_{c}=-\frac{1}{T}\frac{{\Delta H}^{{}^{\circ}}}{R}+\frac{{\Delta S}^{{}^{\circ}}}{R}$$

where K_c_ is the equilibrium constant (also called adsorption distribution coefficient), C_Ae_ (mmol) is the amount of adsorbate adsorbed at equilibrium, and C_e_ is the equilibrium concentration (mmol L^−1^) of MB dye in the solution. Different initial concentrations of MB dye solutions were adsorbed by GDH at 3 different temperatures (298, 318, and 338 K) while keeping other parameters constant to draw the plot [ln (C_Ae_/C_e_) versus C_e_] to estimate K_c_. From the slope, K_c_ can be determined [K = e^(slope)^, where “e” is the mathematical constant approximately equal to 2.718]. The values of ΔH° and ΔS° for adsorption are assumed to be temperature independent and can be calculated from the slope and intercept, respectively, of the plots of lnK_c_ against 1/T [50,53,55].

### 2.8. Adsorption Isotherm Models

The interaction between adsorbate molecules and the adsorbent surface was analyzed using two established models, namely the Freundlich and Langmuir isotherms. Experiments were conducted with different concentrations of MB dyes (5, 10, 20, 30, and 50 ppm). The Langmuir isotherm model and its linearized form were represented by Equations (10) and (11), respectively [49,56].

(10) $$Q_{e}=Q_{\max}\frac{K_{L}C_{e}}{1+K_{L}C_{e}}$$

(11) $$\frac{C_{e}}{Q_{e}}=\frac{1}{Q_{\max}K_{L}}+\frac{C_{e}}{Q_{\max}}$$

where Q_max_ (mmol g^−1^) was the maximum adsorption capacity of dye, and K_L_ (g mmol^−1^), was the Langmuir constant.

Equations (12) and (13) indicate the Freundlich isotherm model and its logarithmic form, respectively,

(12) $$Q_{e}=K_{F}C_{e}{}^{\frac{1}{n}}$$

(13) $$\ln Q_{e}=\ln K_{F}+\frac{1}{n}\ln C_{e}$$

where K_F_ (mmol g^−1^) was an indicative constant related to the adsorption capacity of the adsorbent, and 1/n (0~1) was the adsorption intensity or surface heterogeneity of the adsorbent (GDH) [56].

The adsorbent’s appropriateness for the dye was assessed by means of the separation factor constant (R_L_), derived from the equation Equation (14) as follows. Here, K_L_ represents the Langmuir equilibrium constant (expressed in I/mmol). A value of R_L_ greater than 1.0 indicates unsuitability, R_L_ equal to 1 indicates a linear relationship, an R_L_ value between 0 and 1 suggests suitability, while R_L_ equal to 0 signifies irreversibility [50,52].

(14) $${\;R}_{L}=\frac{1}{1+\left(1+C_{0}K_{L}\right)}$$

### 2.9. Response Surface Methodology

Response surface methodology (RSM) is a technique that establishes a regression model and leverages quantitative data obtained from designed experiments. It is an empirical statistical approach aimed at identifying the most favorable combination of process operational variables. By employing a statistically based experimental design for an adsorption process, RSM can reduce process variability, experimentation time, and costs, all while improving process efficiency. The RSM methodology has found extensive application in chemical engineering and the optimization of sorption processes [57,58].

In this experimental section, we employed the 3-level, 3-factor Box–Behnken design (BBD) to ascertain and validate the parameters affecting the efficiencies of MB dye removal. These parameters, referred to as factors, included time (minutes) (A), initial MB dye concentration (ppm) (B), and GDH weight (g) (C), while keeping other input parameters such as the initial pH of the medium, sample temperature (K), and agitation speed (rpm) constant. The response variable (Y) measured in this study was the MB dye removal rate. The three levels of each factor were coded as −1 (low), 0 (central point), and 1 (high). For a clear representation of the variables and their respective levels, please refer to Table 1, which illustrates the BBD model’s setup. To determine the total number of experimental runs required for this design, the following Equation (15) can be used,

(15) $$N=2k\left(k-1\right)+C_{o}$$

where N is the total number of experimental runs, k is the number of independent variables, and C_o_ is the number of central points [59]. In this research endeavor, a total of 18 experiments were conducted to optimize the impact of three key independent parameters on the efficiencies of MB removal. The experimental error was evaluated using the center points. Prior to conducting the experiments, these parameters and their corresponding ranges were carefully chosen based on insights from previous investigations and pilot studies. For statistical analysis, the Design-Expert software (version 13.0.5.0, Stat-Ease, Inc., Minneapolis, MN, USA) was employed. The obtained results were analyzed using the coefficient of determination (R^2^), Pareto analysis of variance (ANOVA), as well as statistical and response plots. These analytical tools allowed for a comprehensive examination of the data and the extraction of meaningful insights from the experimental outcomes [59,60,61].

### 2.10. Regeneration and Reuse

After MB dye adsorption, the spent GDH was collected using centrifugation at 10,000 rpm for 10 min. Next, 0.5 g of GDH was placed into a 50 mL centrifuge tube, and 40 mL of deionized water (DI water) was added to it. The mixture was then subjected to shaking using a digital orbital shaker TS-500D (Yude Technology Co., Ltd., Xinbei City 23558, Taiwan) at 110 rpm for 30 min to wash the GDH and remove any unbound dye. After washing, the GDH was collected again using centrifugation. For desorption, the adsorbent was treated with 30 mL of methanol (MeOH-99.9%) and placed in an an ultrasonic bath (Elma-Ultrasonic Cleaners-Elmasonic, P 30 H, Elma Schmidbauer GmbH, Gottlieb-Daimler-Straße 17, 78,224 Singen, Germany) for 30 min at a frequency of 37 kHz, maintaining the temperature between 313 and 323 K (40–50 °C), and applying a nominal power of 320 W. Following desorption, the adsorbent was collected once more through centrifugation. The desorption step with MeOH was repeated 3-5 times until the color of the MeOH solvent became colorless, indicating successful desorption of the dye.

Next, the collected GDH underwent an additional cleaning step with 40 mL of DI water and was collected using centrifugation. Subsequently, the GDH was dried overnight at 378 K (105 °C), and the regenerated adsorbent was used in the dye adsorption process to determine the adsorption efficiencies with each repeated use.

## 3. Results and Discussion

### 3.1. Characterization of GDH

Humin is believed to be produced through the condensation of intermediate forms during the dehydration process of sugars. Moreover, all stages of the reaction sequence can be encapsulated within the humin matrix. According to Baccile et al. (2009), approximately 15% of the carbon takes the form of levulinic acid entrapped in hydrothermal carbon spheres [62]. Consequently, it should be feasible to extract these encapsulated compounds using solvents such as water or acetone [62,63]. Additionally, humin is composed of an organic matrix that proves to be unstable during carbonization, leading to anticipated changes. The morphology may vary after carbonization from its original state as GDH. This carbonization step can result in increased surface area and pore volume, the creation of activated carbon, and the decomposition of some organic matter [29]. Hence, comprehending the alterations that occur during this process is crucial [12].

Furthermore, Sayjumpa et al. (2019) found that soil-derived humin carbonized at 773 K (500 °C) exhibited the highest dye removal capacity compared to other carbonized temperatures (300, 700, and 900 °C). For this study, a carbonization temperature of 773 K (500 °C) was selected based on this finding [29].

**Figure 1:**
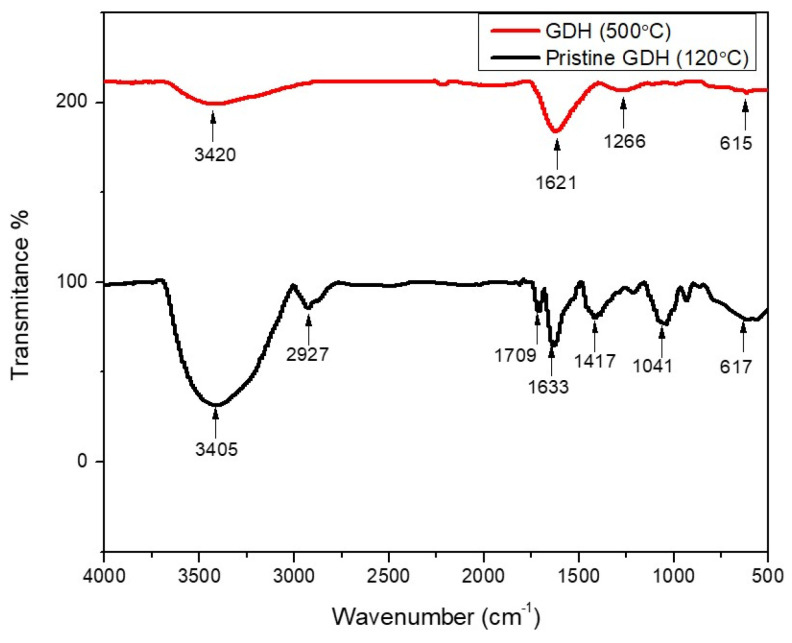
The FT-IR spectra of pristine and carbonized GDH.

The FT-IR spectrum, as shown in Figure 1 and data presented in Table 2, illustrates the distinct bands that indicate the presence of functional groups in GDH. Even though the assignment of the different bands varies across the literature, most authors reported similar spectra. The broad band around 3405/3420 cm^−1^ occurred due to both moistures trapped inside the humin and -OH stretching vibrations of hydroxyl groups in the humin structure. Although this peak was very broad and tall in the pristine GDH, after carbonization, the peak’s broadness and intensity decreased due to the vaporization of moisture. The peaks at 2925 cm^−1^ and 1700 cm^−1^ were assigned to the C-H stretching vibration of methyl and methylene structures (alkyl groups), and C=O stretching vibration of carbonyl groups, respectively. However, these peaks have disappeared from the carbonized GDH due to the decomposition of organic matter at high temperatures. The peaks at 2216 cm^−1^ and 1943 cm^−1^ were due to the O-H stretching vibrations of phenolic groups. The FT-IR spectrum clearly shows the presence of a furanyl structure in humin. For example, the peaks at 1621 cm^−1^ and 1633 cm^−1^ corresponded to C=C stretching vibrations of the furan ring, while the peak at 1417 cm^−1^ was assigned to C-H bending vibrations of the methyl group, respectively. Bands at 1266 cm^−1^, 1213 cm^−1^, and 1041 cm^−1^ were attributed to the C-O stretching vibrations of aliphatic alcohols and ethers. The broad peak in the 800–500 cm^−1^ range revealed the C-H bending vibration of the furan ring [3,8,17,18,49].

**Figure 2:**
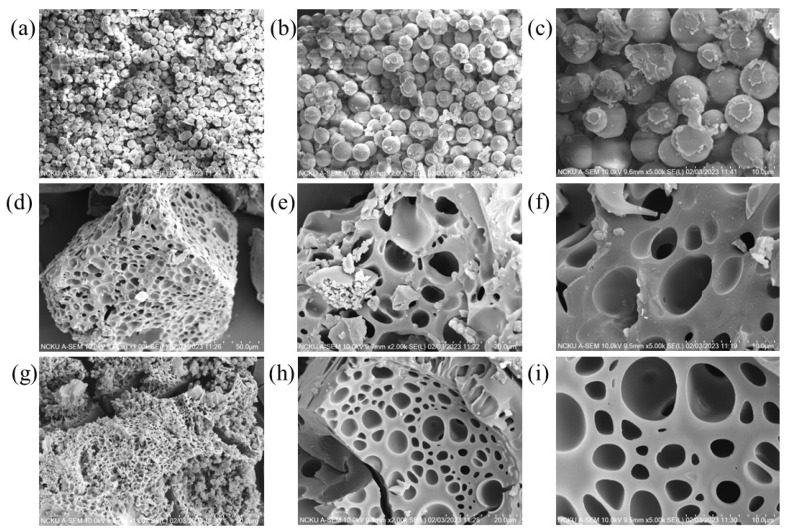
SEM images of the GDH. (**a**) Pristine GDH, 1000×, (**b**) Pristine GDH, 2000× (**c**) Pristine GDH, 5000× (**d**) carbonized GDH, at 500 °C 1000×, (**e**) carbonized GDH at 500 °C 2000×, (**f**) carbonized GDH at 500 °C, 5000×, (**d**) carbonized GDH, at 500 °C 1000×, (**g**) carbonized GDH* at 500 °C 500×, (**h**) carbonized GDH* at 500 °C, 2000×, (**i**) carbonized GDH at 500 °C, 5000×.

Figure 2 presents the SEM images of the GDH samples utilized in this study, magnified at 1000×, 2000×, and 5000×. Figure 2a–c display the SEM images of the pristine GDH synthesized at 120 °C. It is evident that the pristine GDH exhibits sphere-shaped, solid agglomerate structures with smooth surfaces, measuring around 50 μm in size. This size is broader than what has been reported in many previous studies. For instance, Hoang et al. (2015) synthesized humin from D-glucose using the H_2_SO_4_ catalyst at 180 °C for 6 h with a stirring rate of 500 rpm, observing sphere-shaped agglomerate particles sized between 1 and 20 μm. Smaller particles (<3 μm) demonstrated a more defined spherical shape, while larger ones resembled smaller spheres aggregated together [12]. Similarly, Girisuta et al. (2013) and Van Zandvoort et al. (2013) synthesized humin from HMF, glucose, or cellulose using H_2_SO_4_ as the catalyst, yielding particle sizes of around 5–10 μm. According to Van Zandvoort et al. (2013), reaction conditions such as the type of sugar (glucose, fructose, HMF, xylose, etc.), retention time, and agitation speed significantly influenced the morphology of the synthesized humin [3,64].

Figure 2d–f,g,i display the SEM images of carbonized GDH initially synthesized at 120 °C and 160 °C (GDH*), respectively. Following carbonization at 500 °C for 2 h, the GDH’s morphology transforms into a porous structure with pore diameters ranging between 10 and 80 μm. The carbonization process eliminates the spherical shape and results in a high density of pores. In contrast, many previous studies did not clearly indicate changes in particle size and shape after carbonization, even up to 700 °C [3,62,64]. Moreover, only a few studies have shown spheres with cores after carbonization [63].

**Figure 3:**
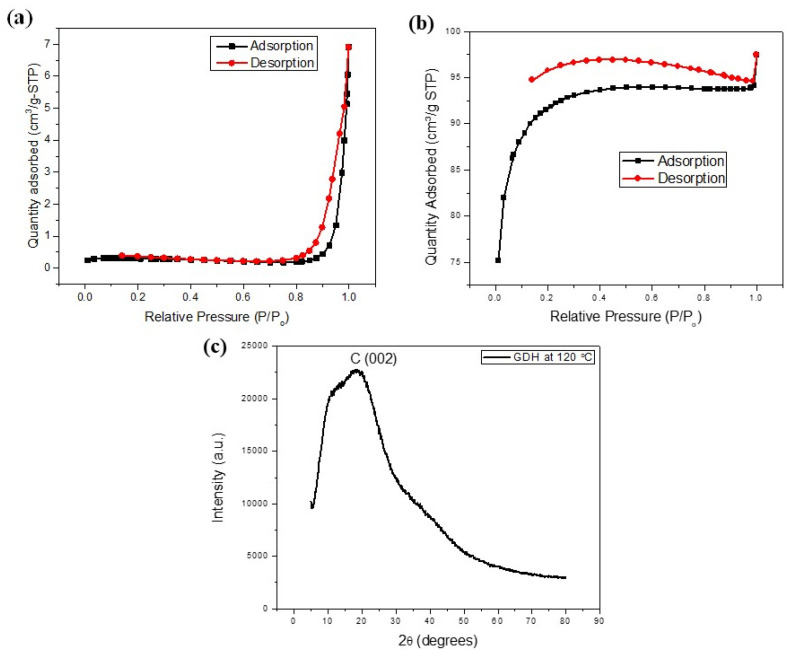
Characterization of GDH. (**a**) BET analysis of pristine GDH- N_2_ adsorption and desorption isotherms, (**b**) BET analysis of carbonized GDH- N_2_ adsorption and desorption isotherms, (**c**) XRD pattern of pristine GDH.

The BET analysis in this study confirmed the presence of high porosity after carbonization. The specific surface area of the pristine GDH was found to be negligible compared to the carbonized GDH, as depicted in Table 3. The nitrogen adsorption and desorption isotherms of the pristine GDH adsorbent exhibited characteristics of the Type II isotherm, indicating its macroporous structure [Figure 3a]. The flatter region in the middle of the isotherm represented the formation of a monolayer. This is the most common isotherm observation when using the BET technique. Nitrogen gas filled the pores at very low pressures, and monolayer formation commenced at the knee, followed by multilayer formation at medium pressure, and capillary condensation at higher pressures. The nitrogen adsorption and desorption isotherms of the carbonized GDH sample exhibit Type I isotherm characteristics [Figure 3b], which result from the interaction between the adsorbent and the adsorbate in micropores of molecular dimensions. Type I is categorized as a pseudo-Langmuir isotherm, as it represents monolayer adsorption. Similar findings were observed in biochar activation processes. For instance, Ghani et al. (2017) synthesized activated carbon utilizing nypa biomass and reported the presence of Type I isotherm characteristics in their results [65].

Table 3 presents the specific surface area, pore size, and pore volume of the pristine GDH synthesized at 120 °C, which were measured as 0.8065 m^2^/g, 56.63 nm, and 0.0093 cm^3^/g, respectively. In addition, Table 3 presents how these values significantly increased to 331.9938 m^2^/g, 36.3267 nm, and 0.094887 cm^3^/g, respectively following the carbonization step. The results clearly show an increase in specific surface area and pore volume in the carbonized GDH. Similar findings have been reported in previous studies as well [19]. For instance, Hoang et al. (2015) observed that at 600 °C and 700 °C, the GDH exhibited significant porosity with specific areas of 463 and 447 m^2^/g, respectively, while the specific area of pristine GDH remained negligible [64]. The surface morphology of GDH underwent slight changes when synthesized at different reaction temperatures. As illustrated in Table 3, increasing the reaction temperature generally led to a slight decrease in the surface area while overall increasing pore size and volume.

XRD patterns of the pristine GDH were obtained within the range of 5° to 80° and are displayed in Figure 3b. Typically, amorphous patterns lack distinct peaks, while crystalline patterns exhibit numerous sharp peaks. The appearance of a broad peak between 18 and 22 degrees in the XRD of the GDH indicates the presence of carbon, likely attributable to the presence of graphite crystallites in the sample. The absence of additional peaks in this diagram confirms the lack of any other X-ray traceable compounds in the GDH sample, reaffirming its amorphous nature. Notably, the XRD patterns do not reveal any traces of humic acid or fulvic acid, suggesting that these compounds have been entirely eliminated from the sample [66,67,68].

### 3.2. Effects of Different Parameters on MB Dye Adsorption

**Figure 4:**
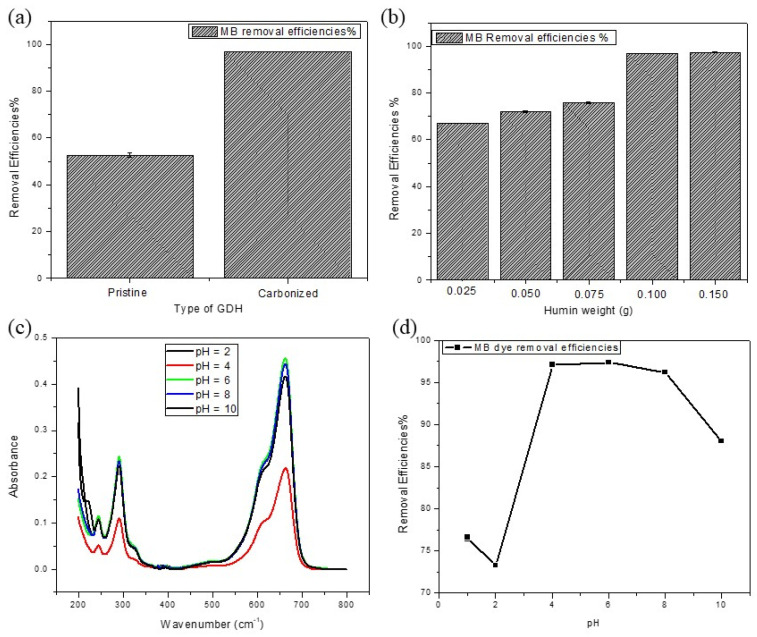
Effects of different parameters towards removal efficiencies of MB dye by the GDH. (**a**) Pristine humin and carbonized humin, (**b**) amount of adsorbent (g), (**c**) λ_max_ of MB dye at different pH, (**d**) pH.

During the initial phase, the reaction parameters and their respective ranges were determined through previous investigations and pilot studies. The efficiency of MB dye removal was found to be 52% for pristine GDH and significantly improved to 97% for carbonized GDH, as shown in Figure 4a.

#### 3.2.1. Effect of Adsorbent Amount

As depicted in Figure 4b, increasing the dosage of GDH adsorbent resulted in a corresponding rise in MB dye removal efficiency. This can be attributed to the increased number of adsorption sites and surface area available for adsorption. When the adsorbent dosage was varied at 0.025, 0.050, 0.075, 0.10, and 0.15 g, the respective removal efficiencies for MB dye were found to be 67.02%, 72.05%, 75.70%, 96.88%, and 97.33%, respectively. However, it is important to note that the MB removal efficiencies gradually approached equilibrium due to the limitation of available MB dye in the reaction medium. Additionally, it should be considered that the adsorption capacity is inversely proportional to the amount of adsorbent used, as an increased amount leads to more activation sites. Therefore, for the subsequent experiment, an adsorbent dosage of 0.1 g was selected.

#### 3.2.2. Effect of PH

The adsorption efficiency of MB is directly affected by the pH of the reaction medium, as pH influences the ionization of MB dye molecules and the charge intensity of the GDH adsorbent [28,65]. Investigation of λ_max_ values of MB dye at different pH levels revealed that λ_max_ remained constant, irrespective of the pH of the medium [Figure 4c]. Furthermore, experimental results presented in Figure 4d showed a gradual increase in MB removal by GDH as the solution pH was raised from 2.00 to 6.00. Notably, at initial solution pH levels of 1.00, 2.00, 4.00, 6.00, 8.00, and 10.00, the corresponding MB removal efficiencies by GDH were 76.56%, 73.26%, 97.00%, 97.37%, 96.19%, and 88.02%, respectively. Humin contains abundant -OH and -COOH functional groups. As the pH of the reaction medium increases, the amount of un-ionized -COOH functional groups in humic substances decreases. This decrease shows a slow trend between pH 1.00 and 4.00, followed by a rapid increase between pH 4.00 and 5.00, and then a return to a gradual increase above pH 5.00. By pH 6.00, nearly all -COOH groups have been ionized [47,69,70]. Since MB dye is a cationic dye with pK_a_ > 14 across the studied pH range, it is attracted to the negatively charged sites on the GDH adsorbent due to electrostatic forces [47,65]. Consequently, when the pH of the medium increases, the number of negatively charged sites on the adsorbent surface also increases, leading to greater adsorption of the positively charged MB dye. The rapid increase in MB dye sorption from pH 2.00 to 6.00 can be attributed to the strong attraction between the positively charged MB dye and the more negatively charged GDH at higher pH values.

However, beyond pH 6.00, the adsorption of MB dye onto GDH experienced a slight decrease with further increase in the pH of the medium. This can be explained by the fact that while the proportion of negatively charged sites on the adsorbent continues to increase with higher pH, the proportion of OH^-^ in the solution also becomes more dominant than H^+^ at pH values higher than 7.00. As a result, there is a competition for the positively charged MB dye between OH^-^ and the negatively charged sites on GDH. This competition leads to a slight reduction in the adsorption of MB dye on GDH with increasing pH of the reaction medium from 6.00 to 10.00 [47].

#### 3.2.3. Effect of Temperature

**Figure 5:**
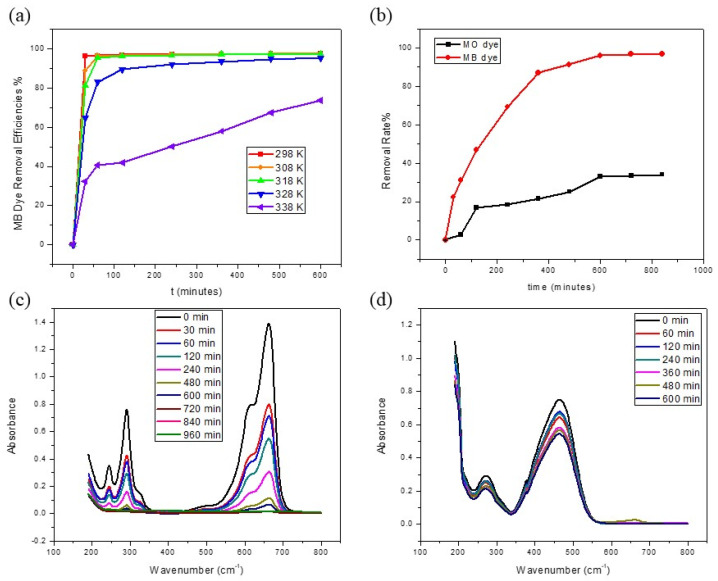
Effects of different parameters towards removal efficiencies of dyes by the GDH. (**a**) MB dye removal plot of different temperatures vs. time, (**b**) MB and MO dyes removal efficiencies vs. time, (**c**) full wavelength scan spectra for MB dye removal at different time, (**d**) full wavelength scan spectra for MO dye removal at different time.

The effect of temperature on the adsorption equilibrium time for the removal of MB dye by GDH is illustrated in Figure 5a. The results show that the adsorption was decreased with an increase in the temperature of the reaction medium from 293 K to 338 K (25 °C to 65 °C). The time taken to reach equilibrium was roughly 360, 480, and 600 min at 298 K (25 °C), 308 K (35 °C), and 318 K (45 °C), respectively, while it had not reached equilibrium even after 600 min at 328 K (55 °C) and 338 K (65 °C). The reason behind this scenario was that the adsorption of MB dye onto GDH is exothermic in nature (heat is released whenever adsorbates are adsorbed on the surface of the adsorbent) [71]. Physisorption involves weak van der Waals’ forces and these forces become weaker when increasing the temperature of the medium. Hence, the adsorption rate decreases whenever the temperature increases. This is also explained by the fact that as the temperature rises, the kinetic energy of adsorbed molecules also rises, allowing them to overcome the electrostatic force of attraction by the adsorbent surface [50].

#### 3.2.4. Comparison of Adsorption of MB and MO Dyes

According to the FT-IR spectrum results and the literature, humin has phenolic -OH and -COOH groups in its structure, and many studies have consistently presented that the -COOH group is considerably more reactive than the -OH group in binding different types of adsorbates [47]. In addition, many biomass-derived activated carbons (biochar) have electronegative surface, and GDH can also be considered a biochar. MB dye is a cationic dye since it would spontaneously interact with the negatively charged parts of the adsorbent through electrostatic interactions. However, since MO dye is an anionic dye, the repulsion forces between anionic MO dye molecules and the negatively charged part of the adsorbent surface of the GDH would occur [28,71]. Therefore, the MB dye has a higher interaction with the GDH surface than the MO dye, since the removal efficiency of MB (97%) is higher than MO’s (33%) removal efficiency. Plots of removal efficiencies of MB and MO dyes vs. reaction time and full wavelength scan UV-visible spectra drawn for absorbance vs. the reaction time are shown in Figure 5b–d, respectively.

### 3.3. Adsorption Kinetics and Thermodynamics

Figure 5b,c illustrate the effect of contact time on dye removal efficiency. It is observed that the removal efficiency increases with time, eventually reaching equilibrium after 600 min of contact time, at which point no further MB dye molecules are adsorbed from the solution. These findings suggest that the adsorption process occurs in three stages: (1) rapid adsorption of MB dye during the initial minutes of interaction, where the abundance of active sites on the GDH surface allows for significant adsorption; (2) with longer contact times, the dye molecules require more time to diffuse into the pores of the adsorbent; and (3) after reaching equilibrium, no further dye adsorption occurs due to saturation of the adsorbent sites on GDH [72].

**Figure 6:**
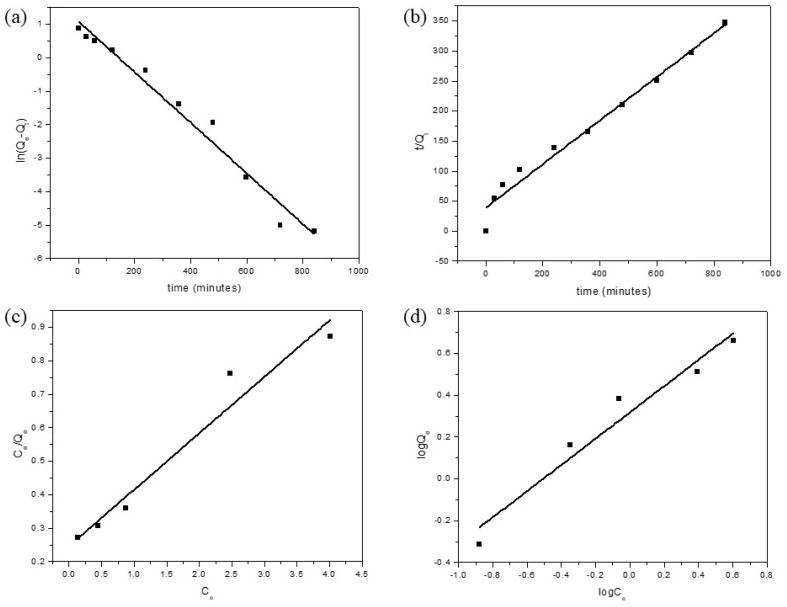
Adsorption kinetic model’s and isotherm model’s fitting results for the removal MB dye by GDH. (**a**) pseudo-first-order model, (**b**) pseudo-second-order, (**c**) Langmuir isotherm model, (**d**) Freundlich isotherm model.

To gain a deeper understanding of the adsorption kinetics, various models have been developed, with the two most common ones being the pseudo-first-order and pseudo-second-order models. The pseudo-first-order model describes a rate of adsorbate removal over time, which is proportional to the variance in saturation and the number of active sites on the adsorbent. This model is suitable for describing the adsorption process and follows a first-order reaction. On the other hand, the pseudo-second-order model explains how the reaction rate is influenced by both the amount of solute adsorbed on the adsorbent’s surface and the amount adsorbed at equilibrium. This model follows a second-order reaction [49,72]. The correlation coefficients (R^2^) of the pseudo-first-order model and the pseudo-second-order model for this experiment were 0.97413 and 0.97543, respectively, indicating that both models have similar R^2^ values [as depicted in Figure 6a,b]. However, when comparing the Q_e_ values obtained from the models with the experimental values (2.42808), the pseudo-first-order model proved to be the most fitting in describing the kinetic mechanism involved in MB dye adsorption by GDH (Table 4). Nevertheless, as shown in Figure 4b, Q_e_ was 6.70 mg/g when the GDH amount was 0.025 g, while other parameters remained constant.

The calculated values of activation energy and the frequency factor for MB dye adsorption were determined to be −94.41 kJ/mol and 8.858 × 10^−16^ g/mg min, respectively. Typically, a low E_a_ value (<42 kJ/mol) indicates a diffusion-controlled process (physical adsorption), while a high E_a_ value (>42 kJ/mol) indicates a chemically controlled process. In this experiment, the E_a_ value was found to be −94.4 kJ/mol (R^2^ = 0.9695), indicating that the rate-determining step is a diffusion-controlled process. The negative activation energy value suggests that increasing the temperature of the reaction medium does not favor MB dye adsorption onto GDH, leading to a decreased probability of colliding particles being captured by the relevant adsorbent. This negative value also indicates the absence of energy barriers in the process [73,74]. In other words, the adsorption process with a negative E_a_ value is exothermic in nature and facilitated by lower temperatures.

The calculated values of thermodynamic parameters ΔG°, ΔH°, and ΔS° are presented in Table 5. The Gibbs free energy of change is used to evaluate the spontaneity and feasibility of adsorption processes. The negative vindicates ΔG° at 298 K (25 °C) and 318 K (45 °C) indicate the spontaneous nature of MB dye adsorption onto the adsorbent, while the positive value at 338 K (65 °C) indicates the non-spontaneous nature of adsorption. The enthalpy change in adsorption experiments provides insights into the nature and mechanism of adsorption processes. A negative ΔH° value indicates an exothermic adsorption process, whereas a positive ΔH° value indicates an endothermic process. The negative value of ΔH° in this experiment suggests that the MB dye adsorption process is exothermic, and the negative value of ΔS° indicates a decrease in randomness at the solid/solution interfaces during adsorption [53,55,75].

### 3.4. Adsorption Isotherm Models

Adsorption isotherm models play a crucial role in understanding the interaction between the adsorbate and the adsorbent used for the removal of organic pollutants. These models describe the relationship between the amount of adsorbate adsorbed by the adsorbent and the amount of adsorbate remaining in the medium. By analyzing these model parameters, we can gain insights into the adsorption mechanism, surface properties, and adsorbent affinity for the adsorbate.

The Langmuir model suggests that monolayer adsorption occurs on a homogeneous adsorbent surface, with the adsorption energy of active sites on the adsorbent being consistently similar. On the other hand, the Freundlich model describes multilayer adsorption on a heterogeneous adsorbent surface, where the adsorption sites of the adsorbent exhibit varying affinities toward the adsorbate. Figure 6c,d present the fitted curves for the Langmuir and Freundlich models in this experiment, and their respective parameters are listed in Table 6. Based on the plotted values and R^2^ values, it is evident that the Langmuir model provides a better fit to the experimental data compared to the Freundlich model. The calculated Q_max_ value of MB dye removal, obtained from the Langmuir plots, was found to be 5.93 mg^−1^, indicating that the adsorption of MB dye onto the adsorbent occurred via monolayer adsorption.

The separation factor (R_L_) is a dimensionless constant used to assess the viability of an adsorption system at various initial dye concentrations. The calculated R_L_ values in this study ranged from 0.028 to 0.185. These R_L_ values exhibited a decreasing trend with increasing initial MB dye concentration and fell within the range of 0 < R_L_ < 1, suggesting a favorable adsorption process [74].

### 3.5. Response Surface Methodology

To optimize the significant variables in this experiment, the Box–Behnken design (BBD) approach was employed. The primary input variables that exert a major influence on the MB dye removal rate are reaction time, initial dye concentration, and adsorbent dose. Statistically designed experiments were conducted to assess the individual and combined effects of these three variables on the removal rate of MB dye by GDH. The complete design matrix and outcomes of the 18 experiments investigated by BBD for MB dye removal are presented in Table 7. The software suggested employing a quadratic model to describe and predict the adsorption process. The final perceived model, expressed in terms of coded factors, is given by Equation (16).

(16) $$Y=+95.63+16.99A-10.96B+17.41C+10.68AB-11.90AC+0.1925BC\;-4.65A^{2}-11.36B^{2}-15.67C^{2}$$

The expressions in the above equation are accompanied by positive and negative signs, indicating their synergistic and antagonistic effects on the response, respectively [59,74]. Reaction time (A), GDH weight (C), and interaction parameters (AB) and (BC) with positive coefficients exhibit a synergistic effect on MB dye removal rate%, while initial MB dye concentration (B), interaction parameter (AC), and quadratic parameters (A^2^), (B^2^), and (C^2^) with negative coefficients have an inhibitory effect on MB dye removal rate%.

Multiple regression analysis was employed to evaluate the response coefficient for the model, and the analysis of variance (ANOVA) technique was used to assess the model’s adequacy and the correlation between input variables and responses. The ANOVA results for MB dye removal rate by GDH are summarized in Table 8. The model F-value of 94.83 indicates its significance. There is only a 0.01% probability that such a large F-value could result from noise. P-values less than 0.0500 suggest significant model terms, and in this case, A, B, C, AB, AC, A^2^, B^2^, and C^2^ are all significant model terms. P-values greater than 0.1000 indicate that the model terms are not significant.

The R^2^ value for Equation (16) was found to be 0.9907, indicating that the model can explain 99.07% of the total deviation in response, with only 0.93% unexplained. Moreover, the predicted R^2^ value (0.8609) closely aligns with the adjusted R^2^ value (0.9803). Typically, a well-fitted model should have an R^2^ value of at least 0.8000. The close proximity of the R^2^ value to 1 and the lower standard deviation (SD) (Table 8) indicate that the developed quadratic model can accurately predict the response within the range of input variables. The predicted R^2^ value of 0.8609 is reasonably consistent with the adjusted R^2^ value of 0.9803 (the difference is less than 0.2).

**Figure 7:**
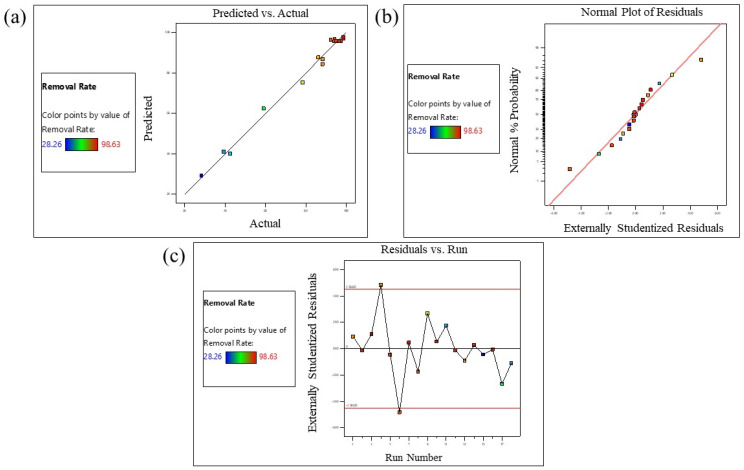
Model validation diagnostic plots for MB dye removal by GDH. (**a**) the plot of predicted values vs. actual values, (**b**) the normal probability plot of studentized residuals, (**c**) studentized residual vs. the run number.

Diagnostic plots, such as predicted vs. actual, residual vs. run, and normal plot vs. residuals, shown in Figure 7a–c, respectively, were also used to assess the model’s fitness [59]. The comparison of model-predicted values based on Equation (16) to experimental values [Figure 7a] demonstrates that the chosen quadratic model effectively investigates the relationship between the input variables (A-reaction time, B-initial concentration of MB dye, and C-GDH weight) and the response (MB dye removal rate %). The normal probability plot in Figure 7b shows that all data points are linearly distributed and closely align with a straight regression line, indicating a good fit of the selected model. The acceptance of this model is further supported by Figure 7c, where the residuals are evenly distributed around the zero line of the graph. The coefficient of variation (CV) value for MB dye removal (Table 7) was calculated to be 3.93%, which is less than 10%, indicating that the model is reproducible and reliable.

**Figure 8:**
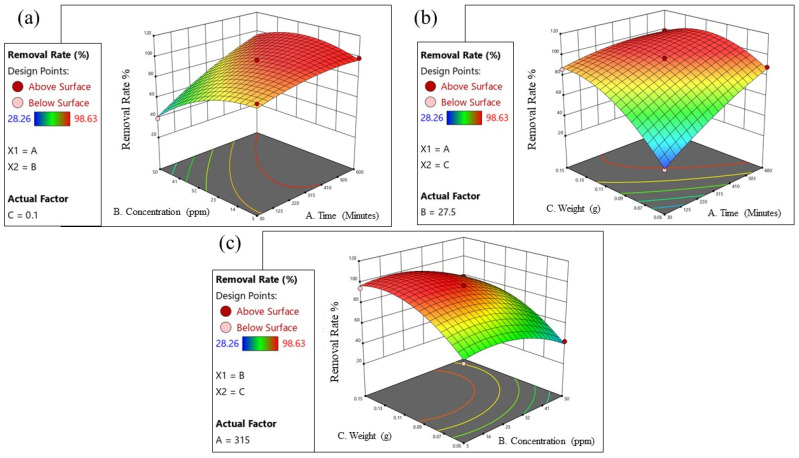
Three-dimensional response surface and contour plots showing combined effects of (**a**) time (A) and initial MB dye concentration (B); (**b**) time (A) and GDH weight (C); (**c**) initial MB dye concentration (B) and GDH weight (C).

The Design-Expert software was utilized to create three-dimensional (3D) surface and contour plots for the model, where two parameters/variables were varied at a time while the other remained constant at the center level (zero level). Figure 8a–c display the 3D surface and contour plots, illustrating the interaction effects of process parameters/variables on the removal rate.

Figure 8a demonstrates the interaction effect of reaction time (A) and MB dye initial concentration (B) while maintaining GDH weight (C) at the zero level. It shows that the removal rate decreased with increasing initial dye concentrations, despite an increase in MB adsorption capacity. The higher adsorption capacity of MB at higher initial concentrations can be attributed to increased contact of adsorbent sites with MB. However, the reduction in dye removal rate with increasing initial MB concentration is primarily due to the limitation of the adsorbent in the medium. The removal rate of MB by GDH increases over time, reaching equilibrium after 600 min of reaction time. The rapid adsorption of MB during the initial minutes of interaction is a result of the higher number of available adsorption sites on the surface of the adsorbent compared to the occupied sites. As the reaction time is extended, the MB molecules require more time to diffuse into the pores of the adsorbent. The optimal reaction time for MB dye removal was found to be 410 min. Figure 8b illustrates the interaction effect of reaction time (A) and GDH weight (C) while maintaining the initial MB dye concentration (B) at the zero level. Increasing both the adsorbent dosage of GDH and the reaction time led to an increase in MB removal rate%. This can be attributed to the higher number of adsorption sites and increased surface area of the GDH adsorbent, as well as the extended duration for adsorption. Figure 8c demonstrates the interaction effect of GDH weight (C) and MB dye concentration while keeping the reaction time (A) at the zero level. At higher MB dye concentrations, the MB removal rate decreased due to the limitation of the GDH adsorbent in the medium. It is evident that the interaction effect between GDH weight and MB dye concentration significantly influences the MB dye removal rate.

**Figure 9:**
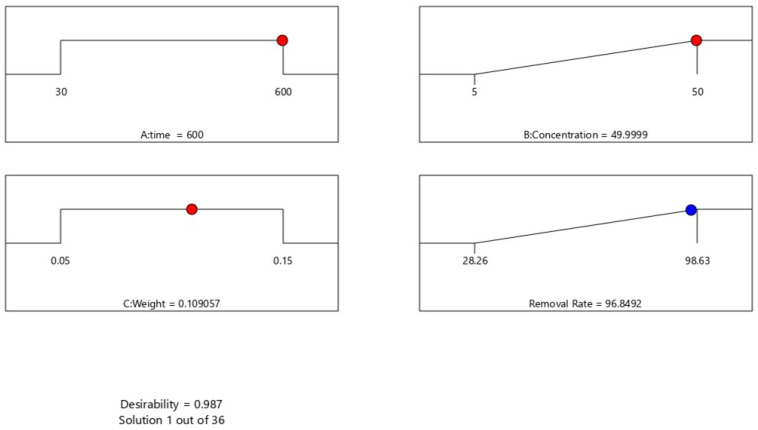
Desirability ramp for numerical optimization of MB dye removal rate.

The desirability function, ranging from 0.00 (undesirable) to 1.00 (highly desirable), was employed to optimize the process parameters/variables and achieve maximum MB dye removal [59]. Numerical optimization was conducted to determine the best combination of input variables that would result in the highest MB dye removal rate. Reaction time and GDH weight were set as “in range,” the initial MB dye concentration was set to “maximum,” and the target was set to “maximum” in order to obtain the most favorable response for MB dye removal, as depicted in Figure 9. The experimental parameters were then verified using the conditions predicted by the BBD model. The optimal parameters predicted by the BBD model were a reaction time (A) of 600 min, an initial concentration of MB dye (B) of 49.99 ppm, and a GDH weight (C) of 0.11 g. The desirability of these predicted values was 98.7% (0.987). The optimized MB dye removal rate projected by the BBD model was 96.85%, which closely matched the experimentally measured value of 92.49%.

### 3.6. Regeneration and Reuse

**Figure 10:**
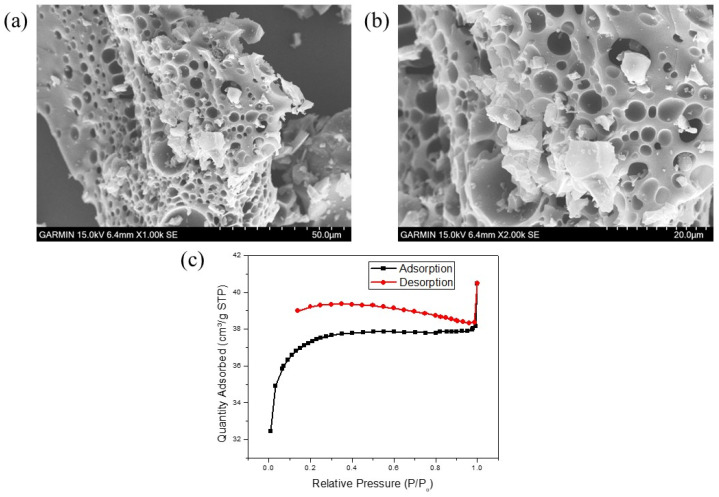
Characteristics of regenerated carbonized GDH, (**a**) SEM image of regenerated GDH 1000×, (**b**) SEM image of regenerated GDH 2000×, (**c**) BET analysis of GDH- N_2_ adsorption and desorption isotherms.

Apart from having excellent adsorption efficiency for dyes, a good adsorbent should also possess the ability to be regenerated and reused. If the adsorbent cannot be effectively reused or if its adsorption efficiency significantly decreases after repeated use, it can lead to secondary environmental pollution and impose additional costs for industrial applications [49]. Methanol, a polar organic solvent commonly used to dissolve various organic compounds, including dyes such as MB, has the capability to effectively solubilize MB molecules, ensuring their uniform dispersion in the solution. Following two dye adsorption-desorption cycles, spent GDH adsorbent was regenerated using MeOH, and the regenerated GDH underwent characterization through BET analysis and SEM analysis to assess any alterations compared to the original carbonized GDH. Figure 10a,b displays SEM images of the regenerated GDH samples, magnified at 1000× and 2000×. As seen in Figure 10a,b, the presence of porous structure in the GDH is evident, and some GDH particles appear to be damaged on the surface. This damage can be attributed to the agitation of the magnet during the MB dye adsorption and during the sonication in the regeneration process. As illustrated in Table 9, the specific surface area, pore size, and pore volume of the regenerated GDH were measured and recorded as 131.01 m^2^/g, 4.80 nm, and 0.05 cm^3^/g, respectively. These results indicate a reduction in surface area, pore size, and pore volume due to the reuse of the adsorbent, which lead to a decrease in its adsorption capability over repeated cycles. Moreover, the nitrogen adsorption and desorption isotherms of the regenerated GDH sample exhibit Type I isotherm characteristics, similar to the initial carbonized GDH, confirming the presence of micropores with molecular dimensions in the regenerated GDH [Figure 10c].

**Figure 11:**
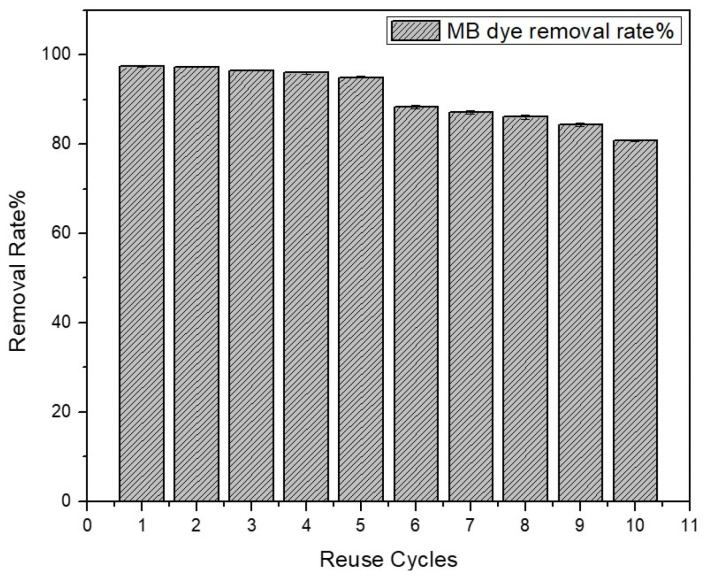
Reuse cycles of regenerated GDH.

As shown in Figure 11, the MB dye removal rate by GDH experienced a slight reduction after each regeneration and reuse cycle. However, even after multiple cycles, it still maintained a remarkably high removal rate compared to the initial use. The MB removal rate remained above 80% even after the tenth cycle. This demonstrates the reusability of the GDH adsorbent after regeneration, resulting in reduced waste generation and offering a cost-effective solution for wastewater treatment.

### 3.7. Literature Comparison

Table 10 presents a comparative analysis of the results obtained in this study for MB dye removal using the byproduct GDH and previously published data. It is noteworthy that only three studies on dye adsorption by humin materials were identified, and all of them utilized humin derived from soil. This research represents the first instance of employing humin derived from the biorefinery process for dye removal from wastewater.

Sayjumpa et al. (2019) utilized humin obtained from Thai leonardite (derived from a lignite mine) for MB dye removal. They achieved a 90% removal efficiency from 25 ppm dye solutions using 1 g of humin after 5 min of contact time and centrifugation at 3000 rpm. It is important to note that the contact time was shorter in their study, and the adsorbent weight was 10% higher compared to our investigation. In our study, after conducting experiments with the optimal parameters obtained from the BBD model, we achieved a 92% removal of MB dye using 0.11 g of GDH in 600 min from a 50-ppm solution. Santosa et al. (2019) employed peat soil humin for MB and p-nitrophenol (p-NP) sorption, yielding adsorption capacities of 70.6 and 36.6 mg/g, respectively. However, their investigation did not provide data on removal efficiency, and the humin weight used was 0.05 g, with dye concentrations at 10 ppm and a contact time of 120 min. Another study by Jesus et al. (2010) involved the use of 0.2 g of peat soil humin to remove 50 ppm starting concentrations of Reactive Orange 16 (RO-16) and Reactive Red 120 (RR-120) in 90 min. The removal efficiencies achieved were 81.4% and 66.8% for RO 16 and RR 120, respectively [29,47,48].

## 4. Conclusions

The isomerization of D-glucose into D-fructose is a crucial industrial conversion with various applications in the food industry, such as in high fructose corn syrup, and as a key intermediate step in producing platform chemicals such as 5-HMF, FDCA, and LA. However, this process leads to the formation of a significant byproduct called humin. In this context, it would be advantageous to utilize GDH as an economical and environmentally friendly adsorbent to remove organic dyes from aqueous solutions. GDH was obtained through the reaction of D-glucose with an AA catalyst in a DES medium, followed by carbonization at 500 °C for 2 h. Cationic MB dye and anionic MO dye were selected as adsorbates in the aqueous medium. The morphology of pristine GDH was altered after the carbonization step, resulting in increased surface area and pore volume, transforming it into activated carbon. The experimental results revealed that the MB removal efficiency of carbonized GDH was higher compared to pristine GDH. Various factors, including the amount of adsorbent, initial MB concentration, reaction temperature, reaction time, and pH of the medium, also influenced the dye removal efficiency. GDH exhibited better removal efficiency for the cationic MB dye compared to the anionic MO dye. Temperature measurements indicated that the MB dye adsorption process was exothermic. The process followed a pseudo-first-order kinetic model, while the Langmuir isotherm provided a comprehensive explanation of the adsorption behavior. The calculated E_a_ value suggested that the rate-determining step of adsorption was diffusion-controlled. To optimize the process, response surface methodology and ANOVA approaches were employed. The significant F-value obtained from the ANOVA technique indicated that the model was meaningful, and P-values less than 0.0500 indicated the significance of model terms. Upon implementing the optimal parameters suggested by the BBD model, the experimental MB dye removal by GDH was found to be in excellent agreement with the predicted value. Notably, GDH demonstrated enhanced removal effectiveness even after regeneration for multiple cycles, particularly after the tenth adsorption cycle, affirming its potential as a green adsorbent for cationic dye removal from wastewater. This highlights the economic and environmental feasibility of utilizing GDH in the entire biomass conversion process.

## Figures and Tables

**Table 1:** Independent variables and their coded levels of Box–Behnken design for MB dye removal by GDH.

Variables	Code	Units	Coded Variables Levels		
			−1	0	+1
Time	A	minutes	30	315	600
Concentration	B	ppm	5	27.5	50
Weight	C	g	0.05	0.1	0.15

**Table 2:** Peak assignments of the FTIR spectra of glucose-derived humin.

Wave Number/cm^−1^	Infrared Adsorption	Functional Groups and Structures
3405, 3420	O-H stretching	Hydroxyl group
2927	C-H stretching	Methyl and methylene structures
2216, 1943	O-H stretching	Phenolic group
1709	C=O stretching	Carbonyl group
1621, 1633	C=C stretching	Furan ring
1417	C-H bending	Methyl group
1266, 1213, 1041	C-O stretching	Aromatic ethers and aliphatic alcohols
800–500 range	C-H bending	Furan ring

**Table 3:** Analysis of specific surface area and porosity of GDH.

Temperature/°C	BET Surface Area/(m^2^/g)	Pore Volume/(cm^3^/g)	Pore Size/(nm)
80	2.7672	0.0177	64.11
100	0.1513	0.0012	69.13
120	0.8065	0.0093	56.63
140	0.5126	0.0136	71.58
160	0.6953	0.0148	75.84
**After Carbonization**	331.9938	0.0949	36.33

**Table 4:** Kinetic parameters for the pseudo-first order model and pseudo-second order Model.

**Pseudo-First Order Model**		
**k_1_ (min^−1^)**	**Q_e_ (mg/g)**	**R^2^**
0.00757	2.42245	0.97413
**Pseudo-Second Order Model**		
**k_2_ × 10^−4^** **(g/mg min)**	**Q_e_ (mg/g)**	**R^2^**
418.145	0.02568	0.97543

**Table 5:** Thermodynamic parameters for MB dye adsorption onto GDH.

Temperature/K	ΔG° (kJ/mol)	ΔH° (kJ/mol)	ΔS° (kJ/mol K)
298	−94.3118	−1670.28	−5.23782
318	−37.8638		
338	105.5307		

**Table 6:** Parameters for Langmuir Isotherm and Freundlich Isotherm Models.

**Langmuir Isotherm Model**		
**Q_max_**	**K_L_**	R^2^
5.93275	0.68219	0.94306
**Freundlich Isotherm Model**		
**n**	**K_F_**	R^2^
1.555932	2.0804	0.9416

**Table 7:** Experimental design matrix for MB dye removal by GDH adsorbent and comparison of actual and predicted values using Box–Behnken design.

	VARIABLES			RESPONSE	
Run	Time (A)	Concentration (B)	Weight (C)	MB Removal Rate%	
				Actual Value	Predicted Value
1	600	27.5	0.05	88.26	86.78
2	315	27.5	0.1	95.23	95.63
3	600	5	0.1	98.63	96.89
4	30	5	0.1	88.24	84.27
5	315	27.5	0.1	94.21	95.63
6	600	50	0.1	92.36	96.33
7	600	27.5	0.15	98.56	97.81
8	315	5	0.15	94.28	96.77
9	315	50	0.15	78.46	75.24
10	315	27.5	0.1	97.25	95.63
11	315	50	0.05	42.53	40.04
12	315	27.5	0.1	95.19	95.63
13	30	27.5	0.15	86.15	87.63
14	315	27.5	0.1	96.48	95.63
15	30	27.5	0.05	28.26	29.01
16	315	27.5	0.1	95.42	95.63
17	315	5	0.05	59.12	62.34
18	30	50	0.1	39.25	40.99

**Table 8:** Statistical technique ANOVA and model summary statistics for response surface quadratic model for MB dye removal rate by GDH.

Source	Sum of Squares	Degree of Freedom	Mean Square	F-Value	*p*-Value	Comment
Model	8748.48	9	972.05	94.83	<0.0001	significant
A-time	2308.94	1	2308.94	225.26	<0.0001	
B-Concentration	960.75	1	960.75	93.73	<0.0001	
C-Weight	2424.86	1	2424.86	236.57	<0.0001	
AB	456.25	1	456.25	44.51	0.0002	
AC	566.20	1	566.20	55.24	<0.0001	
BC	0.1482	1	0.1482	0.0145	0.9072	
A^2^	94.35	1	94.35	9.21	0.0162	
B^2^	563.13	1	563.13	54.94	<0.0001	
C^2^	1071.83	1	1071.83	104.57	<0.0001	
**Residual**	82.00	8	10.25			
Lack of Fit	76.24	3	25.41	22.06	0.0026	significant
Pure Error	5.76	5	1.15			
**Cor Total**	8830.48	17				
**Response (Y)**	**SD**	**CV**	**R^2^**	**Adj. R^2^**	**Pred. R^2^**	**AP**
**MB Removal rate%**	3.20	3.93	0.9907	0.9803	0.8609	28.8300

**Table 9:** Analysis of specific surface area and porosity of regenerated carbonized GDH.

Sample Name	BET Surface Area/(m^2^/g)	Pore Volume/(cm^3^/g)	Pore Size/(nm)
Regenerated carbonized GDH	113.01	0.05	4.80

**Table 10:** Comparison of dye adsorption data by humin.

No.	Adsorbent	Name of the Dye and Concentration	Adsorbent Weight/(g)	Time/(min)	Removal Efficiency	Reference
**1**	Leonardite -humin	MB–25 ppm	1	5	90%	[29]
**2**	Peat soil -humin	MB–10 ppm p-NP–10 ppm	0.05	120	N/A	[47]
**3**	Peat soil -humin	RO 16–50 ppm RR 120–50 ppm	0.2	90	RO 16–81.4% RR 120–66.8%	[48]
**4**	Glucose -derived humin (GDH)	MB dye–25 ppm	0.1	600	97%	This work

N/A = Not Available.

## Data Availability

The data presented in this study are available on request from the corresponding author.
